# Supplementary material for: The Systems Biology Research Tool: evolvable open-source software
Source: BMC Syst Biol. 2008 Jun 29;2:55. doi: 10.1186/1752-0509-2-55 (PMC2446383; doi:10.1186/1752-0509-2-55)
Supplement: Additional file 1 — SBRT Archive. An archive of the current version of the Systems Biology Research Tool. [file 1752-0509-2-55-S1.zip › sbrt-1.4.0/doc/developers_guide/api/sbrt/kernel/fba/proc/CvObjectiveFunctionProcess.html]

CvObjectiveFunctionProcess


|  |  |  |
| --- | --- | --- |
| **Overview** \| **Package** \| **Class** \| **Use** \| **Tree** \| **Deprecated** \| **Index** \| **Help** | |  |
| **PREV CLASS**   **NEXT CLASS** | **FRAMES**    **NO FRAMES**     **All Classes** |  |
| SUMMARY: NESTED \| FIELD \| CONSTR \| METHOD | DETAIL: FIELD \| CONSTR \| METHOD |  |


---


## sbrt.kernel.fba.proc Class CvObjectiveFunctionProcess

```
java.lang.Object
  sbrt.kernel.fba.proc.FbaProcess<FbaOptComputer.Results>
      sbrt.kernel.fba.proc.CvObjectiveFunctionProcess
```

**All Implemented Interfaces:**: KernelProcess<FbaOptComputer.Results>, MonitoredProcess<FbaOptComputer.Results>

---

``` public final class CvObjectiveFunctionProcess extends FbaProcess<FbaOptComputer.Results> implements MonitoredProcess<FbaOptComputer.Results> ```

This class is used to perform constraint-variation objective function
experiments.

**Author:**
:   This class was written and documented by Jeremiah Wright while in the
    Wagner lab.

---

**Constructor Summary**

`CvObjectiveFunctionProcess(FbaOptComputer computer)`
  
          Constructs a new constraint-variation objective function experiment.


| **Method Summary** | |
| --- | --- |
| `Fluxome` | `getFluxome()`             Returns the fluxome used in this experiment. |
| `OptimizationSense` | `getOptimizationSense()`             Returns the current optimization sense. |
| `ProgressMonitor` | `getProgress()`             Returns a progress monitor for this experiment. |
| `void` | `run(ResultsManager<FbaOptComputer.Results> resultsManager)`             Runs this process and adds the generated results to the provided results manager via its `ResultsManager.addData(Object)` method. |
| `void` | `setConstraintSource(java.lang.Iterable<FluxConstraints> constraintSource)`             Sets the constraint variation input source. |
| `void` | `setObjectiveFunctionSource(java.lang.Iterable<? extends MathExpr<java.lang.String>> objectiveSource)`             Sets the objective function input source. |
| `void` | `setOptimizationSense(OptimizationSense sense)`             Sets the optimization sense to the provided value. |

**Methods inherited from class sbrt.kernel.fba.proc.FbaProcess**

`toString`

**Methods inherited from class java.lang.Object**

`clone, equals, finalize, getClass, hashCode, notify, notifyAll, wait, wait, wait`

**Constructor Detail**

### CvObjectiveFunctionProcess

```
public CvObjectiveFunctionProcess(FbaOptComputer computer)
```

:   Constructs a new constraint-variation objective function experiment.

    **Parameters:**: `computer` - the computer with which all results will be computed.


**Method Detail**

### getFluxome

```
public Fluxome getFluxome()
```

:   Returns the fluxome used in this experiment.

    :   **Specified by:**: `getFluxome` in class `FbaProcess<FbaOptComputer.Results>`
    :   **Returns:**: the fluxome used in this experiment.

---


### getOptimizationSense

```
public OptimizationSense getOptimizationSense()
```

:   Returns the current optimization sense.

    :   **Returns:**: the current optimization sense.

---


### setOptimizationSense

```
public void setOptimizationSense(OptimizationSense sense)
```

:   Sets the optimization sense to the provided value.

    :   **Parameters:**: `sense` - an optimization sense.

---


### setObjectiveFunctionSource

```
public void setObjectiveFunctionSource(java.lang.Iterable<? extends MathExpr<java.lang.String>> objectiveSource)
```

:   Sets the objective function input source.

    :   **Parameters:**: `objectiveSource` - a source of objective functions whose optimal values are to be computed.

---


### setConstraintSource

```
public void setConstraintSource(java.lang.Iterable<FluxConstraints> constraintSource)
```

:   Sets the constraint variation input source.

    :   **Parameters:**: `constraintSource` - a source of constraint sets whose effect upon application is to be computed.

---


### getProgress

```
public ProgressMonitor getProgress()
```

:   Returns a progress monitor for this experiment.

    :   **Specified by:**: `getProgress` in interface `MonitoredProcess<FbaOptComputer.Results>`
    :   **Returns:**: a progress monitor for this experiment.

---


### run

```
public void run(ResultsManager<FbaOptComputer.Results> resultsManager)
```

:   Runs this process and adds the generated results to the provided results
    manager via its `ResultsManager.addData(Object)` method.

    First, a set of flux constraints is retrieved from the constraint source
    and applied. Then an objective function is retrieved from the objective
    source. The optimal value is computed and the results are added to the
    results manager.
    Each objective function is iterated through in the same fashion. When
    the end of the objective source is reached, the flux constraints are
    restored to their original value and `resultsManager.breakPoint()`
    is called. At this point, the next set of flux constraints is retrieved
    from the constraint source. The entire process is repeated until the end
    of the constraint source is reached.

    :   **Specified by:**: `run` in interface `KernelProcess<FbaOptComputer.Results>`
    :   **Parameters:**: `resultsManager` - the results manager to which the results will be added.


---


|  |  |  |
| --- | --- | --- |
| **Overview** \| **Package** \| **Class** \| **Use** \| **Tree** \| **Deprecated** \| **Index** \| **Help** | |  |
| **PREV CLASS**   **NEXT CLASS** | **FRAMES**    **NO FRAMES**     **All Classes** |  |
| SUMMARY: NESTED \| FIELD \| CONSTR \| METHOD | DETAIL: FIELD \| CONSTR \| METHOD |  |


---
